# C1 Esterase Inhibitor Reduces Lower Extremity Ischemia/Reperfusion Injury and Associated Lung Damage

**DOI:** 10.1371/journal.pone.0072059

**Published:** 2013-08-26

**Authors:** Claudia Duehrkop, Yara Banz, Rolf Spirig, Sylvia Miescher, Marc W. Nolte, Martin Spycher, Richard A. G. Smith, Steven H. Sacks, Robert Rieben

**Affiliations:** 1 Department of Clinical Research, University of Bern, Bern, Switzerland; 2 Graduate School for Cellular and Biomedical Sciences, University of Bern, Bern, Switzerland; 3 Institute of Pathology, University of Bern, Bern, Switzerland; 4 CSL Behring AG, Bern, Switzerland; 5 CSL Behring GmbH, Marburg, Germany; 6 MRC Centre for Transplantation, Division of Transplantation Immunology and Mucosal Biology, King's College London School of Medicine at Guy's, London, United Kingdom; Université Libre de Bruxelles, Belgium

## Abstract

**Background:**

Ischemia/reperfusion injury of lower extremities and associated lung damage may result from thrombotic occlusion, embolism, trauma, or surgical intervention with prolonged ischemia and subsequent restoration of blood flow. This clinical entity is characterized by high morbidity and mortality. Deprivation of blood supply leads to molecular and structural changes in the affected tissue. Upon reperfusion inflammatory cascades are activated causing tissue injury. We therefore tested preoperative treatment for prevention of reperfusion injury by using C1 esterase inhibitor (C1 INH).

**Methods and Findings:**

Wistar rats systemically pretreated with C1 INH (n = 6), APT070 (a membrane-targeted myristoylated peptidyl construct derived from human complement receptor 1, n = 4), vehicle (n = 7), or NaCl (n = 8) were subjected to 3h hind limb ischemia and 24h reperfusion. The femoral artery was clamped and a tourniquet placed under maintenance of a venous return. C1 INH treated rats showed significantly less edema in muscle (P<0.001) and lung and improved muscle viability (P<0.001) compared to controls and APT070. C1 INH prevented up-regulation of bradykinin receptor b1 (P<0.05) and VE-cadherin (P<0.01), reduced apoptosis (P<0.001) and fibrin deposition (P<0.01) and decreased plasma levels of pro-inflammatory cytokines, whereas deposition of complement components was not significantly reduced in the reperfused muscle.

**Conclusions:**

C1 INH reduced edema formation locally in reperfused muscle as well as in lung, and improved muscle viability. C1 INH did not primarily act via inhibition of the complement system, but via the kinin and coagulation cascade. APT070 did not show beneficial effects in this model, despite potent inhibition of complement activation. Taken together, C1 INH might be a promising therapy to reduce peripheral ischemia/reperfusion injury and distant lung damage in complex and prolonged surgical interventions requiring tourniquet application.

## Introduction

Lower extremity ischemia/reperfusion injury (IRI), which may result from thrombotic occlusion, embolism, trauma or surgical intervention through tourniquet application and subsequent restoration of blood flow, is of essential clinical importance. The deprivation of blood and oxygen, termed as ischemia, leads to time-dependent molecular and structural changes of the affected tissue. Complex inflammatory cascades are subsequently activated when blood flow is restored, leading to ischemia/reperfusion injury (IRI). The hypoxic state of ischemia leads to expression of non-muscle myosin heavy chain type II or annexin IV on the cell surface, which function as neo-epitopes for natural antibodies [1],[2]. This immune complex formation already occurs prior to tourniquet release and paves the way for activation of the complement system. Natural antibodies can activate complement via C1q and the classical pathway or via the lectin pathway by binding of mannose-binding lectin (MBL) to carbohydrate structures, particularly on IgM, [3],[4] generating potent anaphylatoxins and ultimately resulting in the formation of a pore and lysis of the cell. The roles of natural antibodies and the complement system in IRI are well established, but the coagulation- and the kinin systems have been shown to be of equal importance [5]. The coagulation system plays a pivotal role in IRI in the intestine, brain, lung and heart [6],[7], [8], [9]. The fact that the complement system may be activated by thrombin, a protease of the coagulation system, highlights the complexity of the inflammatory response in IRI [10]. In a homeostatic situation, the inner lining of blood vessels, the endothelium, maintains an anti-coagulatory and anti-inflammatory environment [11]. This is, amongst others, upheld by the protective layer of the glycocalyx, a negatively charged, tight meshwork of proteoglycans, including heparan sulfate and other glycosaminoglycans and associated plasma proteins. However, during ischemia the glycocalyx may be partially lost [12], [13]. This shedding renders the anti-inflammatory and anti-coagulatory state a pro-inflammatory and pro-coagulatory one and facilitates interaction of leukocytes with the endothelium [14].

The activation of this multifaceted network of cascades in IRI manifests itself in edema formation and muscle necrosis. IRI of the extremities is often accompanied by remote organ damage, affecting organs like the liver, lung, kidney or intestine and may lead to the development of multiple organ dysfunction syndrome [15]. In particular, remote lung damage, which results from the systemic inflammatory response, is a common issue [16]. It has been shown that the expression of pro-inflammatory cytokines is required for remote lung injury, resulting in increased vascular permeability [17].

APT070, also known as Mirococept, is a highly effective complement inhibitor. It is a modified fragment of the complement receptor 1 (CR1) and has binding sites for C3b and also C4b [18]. APT070 consists of the first 3 consensus domains of the human CR1 and a membrane-targeted synthetic peptide, which mediates the binding to phospholipids on the cell surface and therefore protects the cell against complement activation [19]. Beneficial effects of APT070 were shown in our lab in an in vivo study of myocardial infarction by using a closed-chest pig model [20].

C1 esterase inhibitor (C1 INH) is one of the main regulators of the complement system, as it interacts with all three pathways and additionally plays a pivotal role in the coagulation- and kinin systems [21]. Patients deficient in C1 INH suffer from the potentially life-threatening disorder hereditary angioedema (HAE), emphasizing the importance of C1 INH in the healthy organism [22]. HAE patients suffer from edema formation in the upper airways and gastrointestinal tract, [23] mediated by bradykinin, a member of the kinin system that enhances capillary permeability. As C1 INH does not only act on the complement- but also on the coagulation- and the kinin systems, it represents a promising therapeutic option to treat IRI. Positive effects were already shown in IRI of the heart, brain, liver and muscle [24], [25], [26], [27]. We therefore hypothesized, that C1 INH treatment in peripheral IRI would reduce local edema formation as well as lung damage. The effect of exogenous human plasma-derived C1 INH on tourniquet-induced IRI was investigated in a rat hind limb model and the underlying mechanisms of protection were analyzed.

## Materials and Methods

### Animals and housing

All experiments were conducted in accordance with the terms of the Swiss animal protection law and were approved by the animal experimentation committee of the cantonal veterinary service (Canton of Bern, Switzerland) [28]. Male Wistar rats (wild type, bred at the central animal facility, University of Bern) were kept in groups of three in a clear 1500 cm^2^ Euro-standard Type IV S cage (Tecniplast, Buguggiate, Italy) under standard housing conditions with food and water ad libitum. Cages were individually ventilated at 20±2°C and 45–65% relative humidity with a circadian rhythm of 12/12 h. During the light cycle animals were exposed to an intensity of 200 lux. For the experiments, rats weighing between 250 and 350 g were used.

### Reagents

C1 esterase inhibitor (Berinert^®^) as well as the vehicle (10 mg/ml glycine, 2.9 mg/ml sodium citrate, 8.5 mg/ml sodium chloride, pH 7.0) were provided by CSL Behring (CSL Behring GmbH, Marburg, Germany). APT070 was provided by King's College (London, UK) and consists of the first three short consensus repeats of human complement receptor 1. APT070 is modified with a membrane-targeting amphiphilic peptide based on the naturally occurring membrane-bound myristoyl-electrostatic switch peptide [18]. APT070 was provided in a solution of phosphate-buffered saline (PBS, pH 7.4) containing mannitol (50 mg/ml) and arginine (17.4 mg/ml).

### Experimental groups

Rats were divided into five groups. The experimental group (n = 6, C1 INH group) received a dose of 50 IU/kg (50 IU/ml) of human C1 INH. Control group 1 (n = 8, NaCl group) received 1 ml/kg of 0.9% sodium chloride. Control group 2 (n = 7, vehicle group) received 1 ml/kg C1 INH vehicle prior to ischemia. Control group 3 (n = 4, APT070 group) received 9 mg/kg (9 mg/ml) of APT070 before induction of ischemia and control group 4 (n = 4, normal) underwent no intervention.

### Anesthesia and analgesia

Anesthesia was induced with 2.5% isoflurane in oxygen in a box and later maintained by inhalation of 1.5% isoflurane on a nose mask. Analgesia was provided by 0.05 mg/kg of buprenorphine (Temgesic, Reckitt Benckiser, Switzerland AG) injected subcutaneously 30 minutes prior to surgical intervention. The total duration of anesthesia was approximately 6 h after which the rats were allowed to wake up. To provide adequate analgesia for the 24 h reperfusion period buprenorphine injection was repeated when animals were completely awake. After completion of 24 h reperfusion, rats were anesthetized again as described above and sacrificed by exsanguination during organ removal.

### Surgical procedure

For assessment of limb perfusion the fur was completely removed from both hind limbs with an electric shaver. The rats were kept on a heating pad to maintain the body temperature at 37°C. Approximately 30 minutes after induction of anesthesia, the femoral artery and vein were exposed via a groin incision and a tourniquet (standardized weight of 450 g) was placed underneath the femoral vessels to block collateral circulation [29]. The femoral artery was then occluded for 3 h with two microvascular clamps (B1-V, S&T, Neuhausen, Switzerland). Rat hind limbs were not exsanguinated, but a comparable state was achieved by allowing venous return during the entire period of ischemia in order to prevent venous congestion and additional injury through microcirculatory impairment, which would not represent the clinical situation. After 3 h of ischemia the limb was reperfused for 24 h during which the rats were allowed to wake up with appropriate analgesia. At the end of the experiments, tissue samples of both the ischemic as well as the contralateral gastrocnemic muscles as well as the lungs were taken for subsequent analyses.

### Assessment of edema formation

For assessment of edema formation two samples of the gastrocnemic muscle from both legs were taken and immediately weighed to obtain the wet weight. The muscle samples were then dried for 24 h at 80°C after which a constant dry weight was achieved. Subsequently, the wet/dry ratio was calculated.

### Analysis of muscle viability

IRI severely affects muscle viability, which may ultimately result in muscle necrosis. In order to investigate the influence of C1 INH on muscle viability the MTT (3-(4,5-dimethylthiazol-2-yl)−2,5-diphenyltetrazolium bromide, Sigma, St. Louis, USA) assay was performed. MTT is a yellow-colored tetrazolium salt, which is converted to purple colored formazan crystals by metabolically active cells. Muscle samples from the gastrocnemic muscle were taken, washed in PBS, blotted dry and incubated in 0.1 mg MTT/ml PBS in a total volume of 3 ml at 37°C, rotating in the dark for 2 h. Thereafter, muscle samples were blotted dry and incubated in 100% isopropanol at 37°C, rotating in the dark overnight to elute the formazan crystals from the tissue for measurement of the optical density (OD). 200 μl of thus obtained supernatant was measured in a microplate (Nunc, 96 well, maxisorp, transparent, Roskilde, Denmark) with a microplate reader at 560 nm (Ref. 690 nm; Infinite M1000 spectrophotometer, Tecan, Männedorf, Switzerland). After drying the muscle samples at 80°C for 24 hours the OD per mg dry weight was calculated and compared with values of contralateral control legs.

### Histological assessment of damage

For assessment of hemorrhage, total myocyte damage as well as infiltration of neutrophil granulocytes, tissue samples from the gastrocnemic muscle were fixed in 4% formalin for 24–72 h. Thereafter, all samples were embedded in paraffin, cut into 3 μm thick sections and stained with hematoxylin and eosin.

### Immunofluorescence analyses of tissue samples

Immunofluorescence staining using specific antibodies was used to quantify the deposition of IgM (3020-08; Southern Biotech, AL, USA) and IgG (3030-08; Southern Biotech), C1q (A0136, Dako, Baar, Switzerland), MBL (clone 14C3 kindly provided from Prof. G. Stahl, Boston, USA), C4b/c (LSB 4228, LifeSpan BioSciences Inc., Seattle, WA, USA), C3b/c (A0062, Dako) and factor B (341272, Calbiochem, Darmstadt, Germany). Furthermore, we analyzed fibrin deposition (F0111; Dako, Baar, Switzerland), expression of heparan sulfate (HS; 370255, Amsbio, Abingdon, UK), bradykinin receptor b1 (ABR-011, Alomone Labs, Jerusalem, Israel), bradykinin receptor b2 (ABR-012, Alomone Labs) as well as VE-cadherin (sc-6458, Santa Cruz Biotechnology, Inc., Santa Cruz, CA, USA). Tissue samples from the gastrocnemic muscle of both legs and the lung were taken, washed in PBS, blotted dry and embedded in OCT matrix (Tissue-Tek, Sakura Finetek Europe B.V., Leiden, The Netherlands) on dry ice. The samples were immediately stored at −20°C until cryosections were cut. Sections were fixed in acetone and rehydrated in Tris-buffered saline (TBS). Primary antibodies were incubated overnight at 4°C and secondary antibodies were incubated for 1 h at room temperature (RT). Subsequently, slides were mounted and coverslipped. Pictures were taken with a fluorescent microscope (Leica DMI 4000B, Leica Microsystems Schweiz AG, Heerbrugg, Switzerland) and analyzed using Image J (National Institutes of Health, Bethesda, MD, USA) and GraphPad Prism 5 software (GraphPad Software, Inc., San Diego, CA, USA). Endothelial expression of VE-cadherin as well as bradykinin receptor b1 and b2 was analyzed in lung tissue. For this analysis, the inner lining of the vessels was selected by hand, the surface area calculated and the intensity of immunofluorescence measured. Area under the curve values were obtained and divided by the surface area to achieve a final value in intensity per square pixel.

### Assessment of apoptosis using TUNEL

For assessment of apoptosis in muscle and lung tissue a TdT-mediated dUTP nick end labeling (TUNEL) assay (in situ Cell Death Detection Kit, TMR red, Roche, Mannheim, Germany) was used. In brief, cryosections of muscle and lung tissue were fixed in acetone for 5 minutes at RT, washed and permeabilized with 0.1% Triton-X-100 on ice. Sections were incubated with TUNEL reaction mixture for 1 h at 37°C in the dark. After a washing step sections were mounted, coverslipped and analyzed with a fluorescent microscope.

### Analysis of infiltration of myeloperoxidase positive cells in lung tissue

For quantitative analysis of infiltration of myeloperoxidase (MPO) positive cells in lung tissue, embedded and frozen tissue was cut into 5 μm thick sections, fixed in acetone and hydrated in TBS. Tissue sections were stained with an antibody for MPO (A0398, Dako) as well as DAPI (4′,6-diamidino-2-phenylindole) to stain nuclei. Primary antibody was incubated overnight at 4°C and the secondary antibody (C2306, Sigma-Aldrich Chemie GmbH, Buchs, Switzerland) as well as DAPI were incubated for 1 h at RT. MPO positive cells were counted and divided by total number of cells.

### Cytokine/chemokine/growth factor analysis using multiplex array

A multiplex immunoassay consisting of magnetic beads conjugated with a capture antibody specific for a target protein was used to detect an array of cytokines, chemokines, and growth factors (Bio-Plex Pro Rat Cytokine Group I panel, Bio-Rad, Hercules, CA, USA). The assay was performed according to the manufacturer's instructions. Briefly, plasma was diluted 1∶3 and incubated with antibody-coupled magnetic beads. A washing step was followed by incubation with biotinylated detection antibody. After streptavidin-phycoerythrin incubation cytokine/chemokine/growth factor concentrations were measured. Recombinant proteins were used to establish standard curves. Analyte concentrations were calculated using the Bio-Plex Manager Software.

### Statistical analysis

Data are expressed as mean ± standard deviation (SD). Statistical significance was determined by one-way analysis of variance with Dunnett's post-test against NaCl control, using GraphPad Prism 5 software. P values of <0.05 were considered statistically significant. Determination of n-numbers per group was performed without formal power analysis, based on preliminary experiments with C1 INH.

## Results

### Effect of C1 INH treatment on edema formation and muscle viability as well as histological assessment of muscle damage

**Figure 1:**
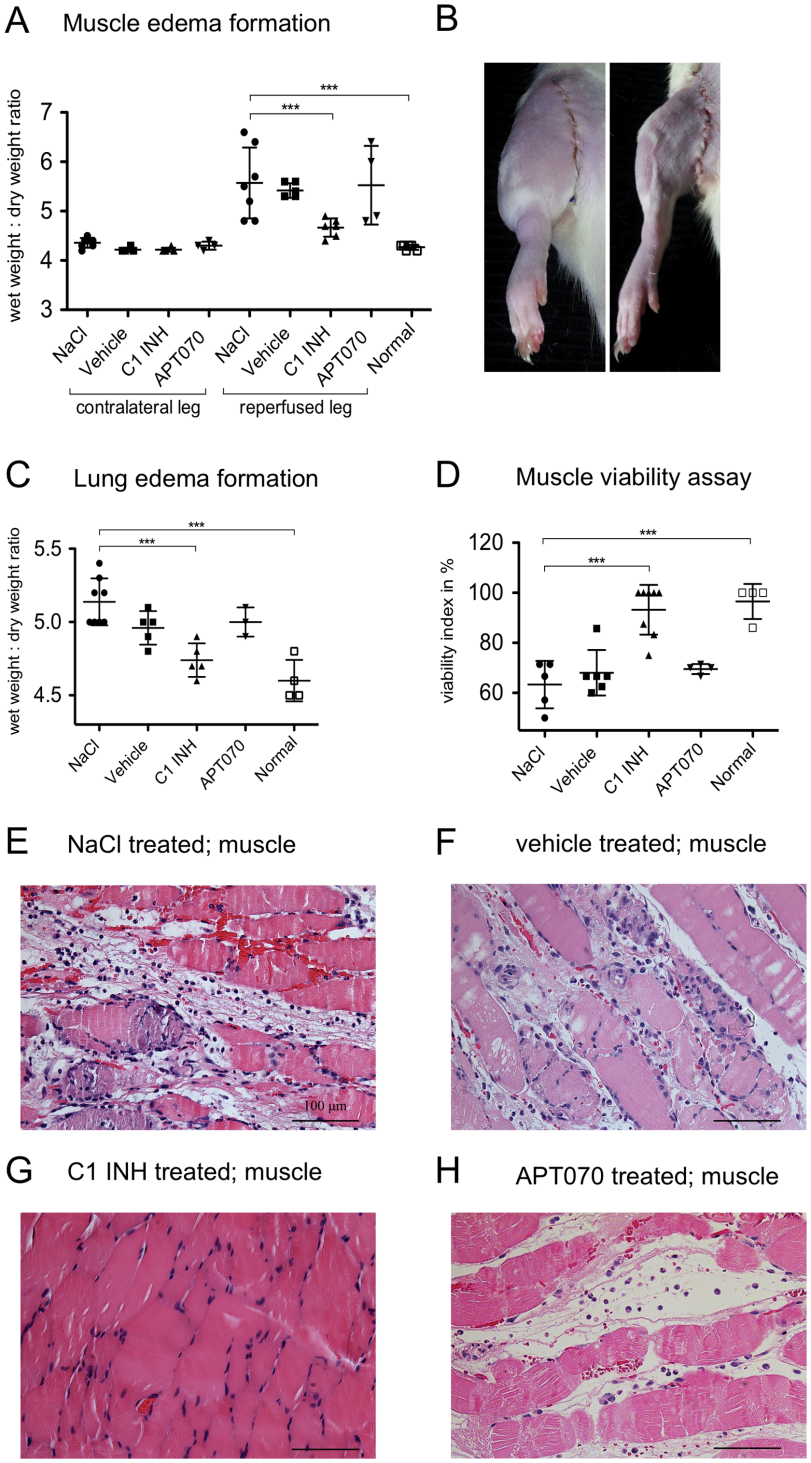
Effect of C1 INH on edema formation, muscle viability and histological assessment of muscle damage. (A) Analysis of edema in the gastrocnemic muscle of both the contralateral- and reperfused legs. NaCl treated rats were compared with C1 INH, APT070 as well as vehicle treated and normal rats. C1 INH treatment reduced muscle wet weight/dry ratio for C1 INH as compared to NaCl controls. (B) Representative images of edema formation after 24 h reperfusion for treatment with NaCl (left) and C1 INH (right). (C) Edema formation in the lung. C1 INH treatment led to a reduced lung wet/dry weight ratio as compared to NaCl controls. (D) Viability of the gastrocnemic muscle as assessed by MTT. C1 INH treatment improved muscle viability as compared with NaCl controls. (**E**–**H**) Hematoxylin/eosin stained histological samples of reperfused gastrocnemic muscle. Representative images are shown for NaCl (**E**) and vehicle (**F**) controls as well as C1 INH (**G**) and APT070 (**H**) treatment. One-way ANOVA followed by Dunnett's post hoc test for significance vs. NaCl controls was used. Error bars indicate mean ± SD. *P≤0.05; **P<0.01; ***P<0.001.

Edema formation in tissue samples was analyzed as wet/dry ratio. Edema was indicated by an increase in wet/dry ratio. C1 INH treatment (ratio 4.6±0.18, Figure 1B right) led to a significant (P<0.001) reduction of fluid accumulation in the gastrocnemic muscle in comparison to NaCl control (5.6±0.71, Figure 1B left). When rats were treated with the complement inhibitor APT070 (5.5±0.79) no attenuation of edema was found (Figure 1A). Furthermore, C1 INH treated rats also showed a significant (P<0.001) reduction in lung edema (4.7±0.11) as compared to NaCl controls (5.1±0.15), whereas APT070 treatment (5.0±0.10) did not lead to reduction of edema formation (Figure 1C). Analysis of muscle viability using the MTT assay showed that C1 INH treatment (viability 93±15.3%) led to a significant increase (P<0.001) of viability in comparison to NaCl control (63±9.4%). Again, this was not the case for APT070 treatment (69±2.0%) (Figure 1D). Histologically, hemorrhage, edema formation and myocyte destruction were apparent in NaCl-, vehicle- and APT070 treated rats, whereas C1 INH treated rats showed only minimal tissue damage (Figure 1E–H).

### Deposition of IgM and IgG in reperfused muscle as well as in lung tissue

**Figure 2:**
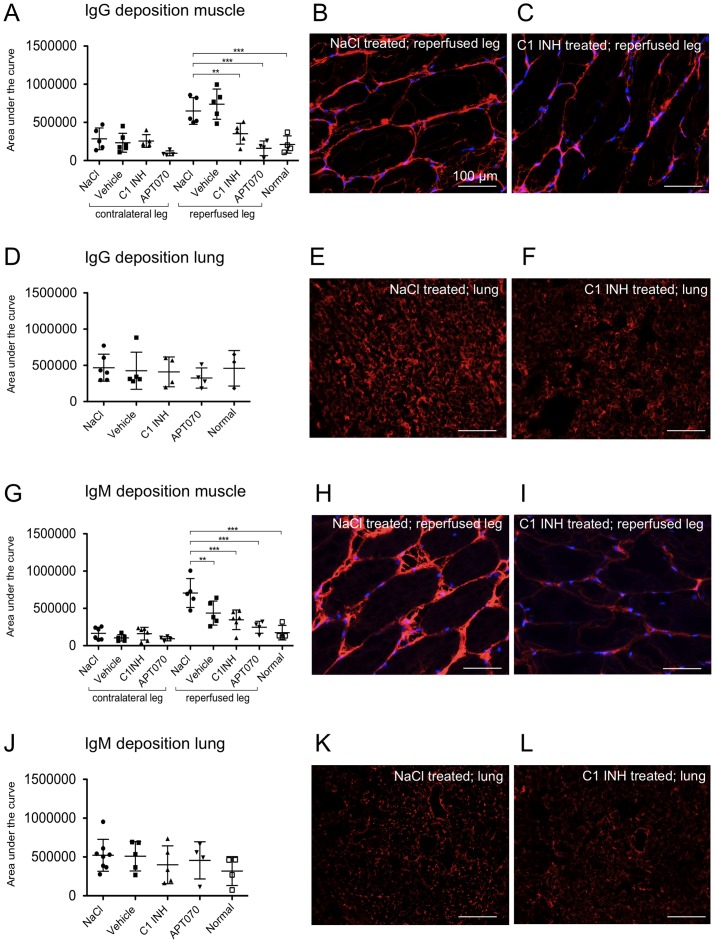
Analysis of deposition of IgM and IgG in muscle as well as in lung tissue. (A, D, G and J) Quantitative analysis of immunofluorescence stainings. (A) Detection of IgG in muscle and (**D**) in lung tissue. (G) Detection of IgM in muscle and (**J**) in lung tissue. (B and C) Representative immunofluorescence images of IgG deposition in muscle and (E and F) in lung tissue of either an NaCl or C1 INH treated rat. (H and I) Representative immunofluorescence images of IgM deposition in muscle and (K and L) in lung tissue of either an NaCl or C1 INH treated rat. IgM as well as IgG detectable in the red channel (CY3), counterstaining with DAPI (blue channel). One-way ANOVA followed by Dunnett's post hoc test for significance vs. NaCl controls was used. Error bars indicate mean ± SD. *P<0.05; **P<0.01; ***P<0.001.

Immunofluorescence stainings were performed to investigate the impact of C1 INH treatment on antibody deposition in muscle and lung tissue. As compared to normal rats no IgG deposits (Figure 2A) or IgM deposits (Figure 2G) were found in contralateral legs. In the reperfused legs high antibody deposition was found for NaCl and vehicle control groups, whereas C1 INH (P<0.01) as well as APT070 (P<0.001) treatment significantly reduced antibody deposition in comparison to NaCl. Representative immunofluorescence images showed an intense staining for IgG (Figure 2B) and IgM (Figure 2H) in reperfused muscles of NaCl treated rats. For C1 INH treated rats reduced deposition was detected (IgG, Figure 2C; IgM, Figure 2I). In lung tissue no differences between groups could be detected (Figure 2D–F and J–L).

### Assessment of deposition of C3b/c and factor B in muscle and lung tissue

**Figure 3:**
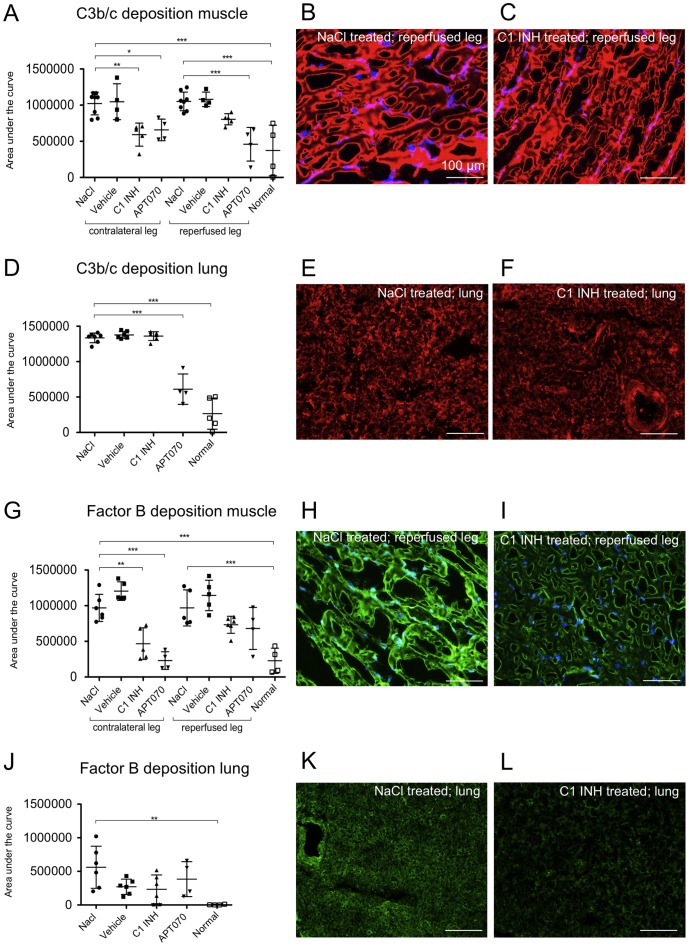
Deposition of C3b/c and factor B in muscle and lung tissue. (A, D, G and J) Quantification data of C3b/c and factor B deposition in muscle and lung tissue, respectively. (B and C) Representative immunofluorescence images of C3b/c deposition in muscle tissue. (**E** and **F**) Representative immunofluorescence images of C3b/c deposition in lung tissue. Counterstaining with DAPI (blue channel, only shown for muscle tissue), C3b/c visible in the red channel (CY3). (**H** and **I**) Representative immunofluorescence images of factor B deposition in muscle tissue. (**K** and **L**) Representative immunofluorescence images of factor B deposition in lung tissue. Counterstaining with DAPI (blue channel), factor B visible in the green channel (Alexa 488). One-way ANOVA followed by Dunnett's post hoc test for significance vs. NaCl controls was used. Error bars indicate mean ± SD. *P<0.05; **P<0.01; ***P<0.001.

Deposition of factor C3, a central component of the complement system, was analyzed using immunofluorescence staining for the C3b/c. High deposition of C3b/c was found in the contralateral as well as in the reperfused muscle tissue and the lung of NaCl as well as vehicle treated rats. C3b/c deposition was significantly reduced by APT070 treatment in both legs (P<0.01) and in the lung (P<0.001), and by C1 INH in the contralateral leg only (P<0.01), but not in the reperfused leg or the lung (Figure 3A–F). Complement factor B, which is specific for alternative pathway activation, was highly deposited in the reperfused and in the contralateral leg as well as in lung of the NaCl control and vehicle groups. C1 INH (P<0.01) as well as APT070 (P<0.001) treatment significantly reduced deposition of factor B in the contralateral leg but not in the lung and reperfused leg (Figure 3G–L).

### Assessment of deposition of C1q, MBL and C4b/c in muscle tissue

**Figure 4:**
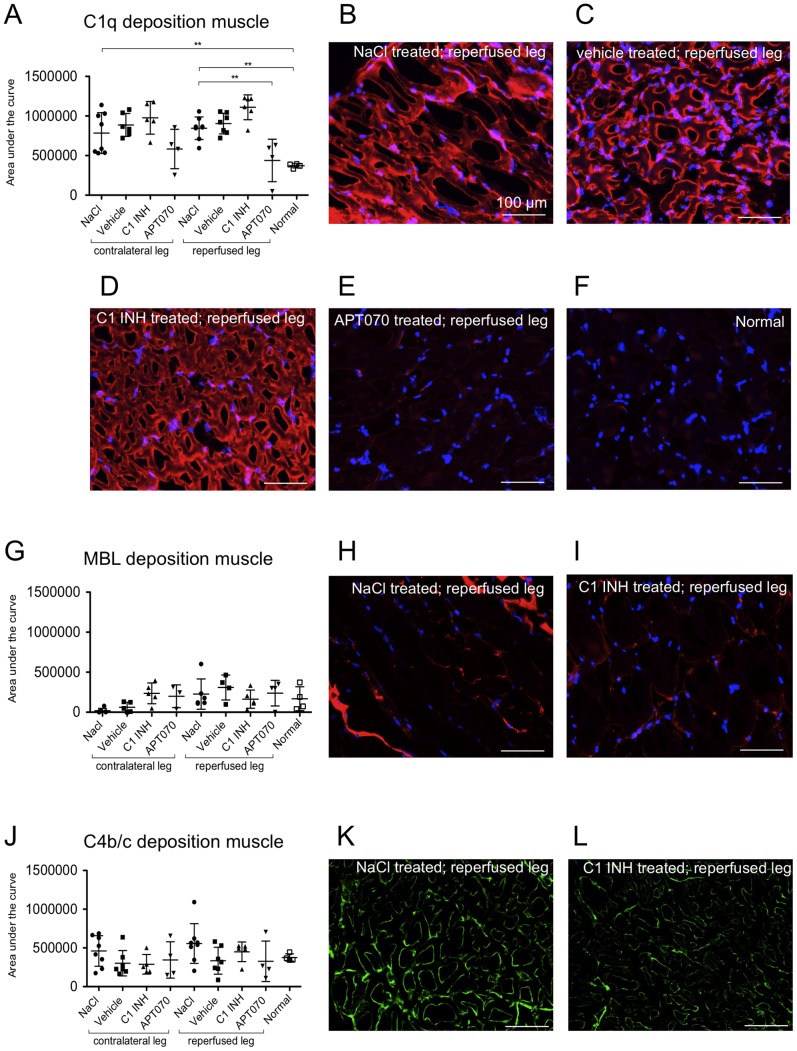
Deposition of C1q, MBL and C4b/c in muscle tissue. (A, G and J) Quantification data of C1q, MBL and C4b/c deposition in muscle tissue. (B–F) Representative immunofluorescence images of C1q deposition depending on treatment. (H and I) Representative immunofluorescence images of MBL deposition in muscle tissue. Counterstaining with DAPI (blue channel), C1q or MBL visible in the red channel (CY3). (K and L) Representative immunofluorescence images of C4b/c in muscle tissue, C4b/c visible in the green channel (Alexa 488). One-way ANOVA followed by Dunnett's post hoc test for significance vs. NaCl controls was used. Error bars indicate mean ± SD. *P<0.05; **P<0.01; ***P<0.001.

To assess deposition of classical- and lectin-pathway specific complement components, stainings for C1q (classical pathway, Figure 4A–F), MBL (lectin pathway, Figure 4G–I) and C4b/c (classical and lectin pathways, Figure 4J–L) were performed. An increased C1q deposition was found for NaCl controls in the gastrocnemic muscle of both legs compared to normal rats (Figure 4A). Enhanced C1q deposition was significantly reduced in the reperfused leg by APT070 but not by C1 INH treatment, whereas no significant differences between groups were detected in the contralateral leg. No inter-group differences were found for deposition of MBL (Figure 4G) as well as C4b/c (Figure 4J) in both reperfused and contralateral legs.

### Impact of C1 INH treatment on fibrin deposition as well as heparan sulfate expression in muscle and lung tissue

**Figure 5:**
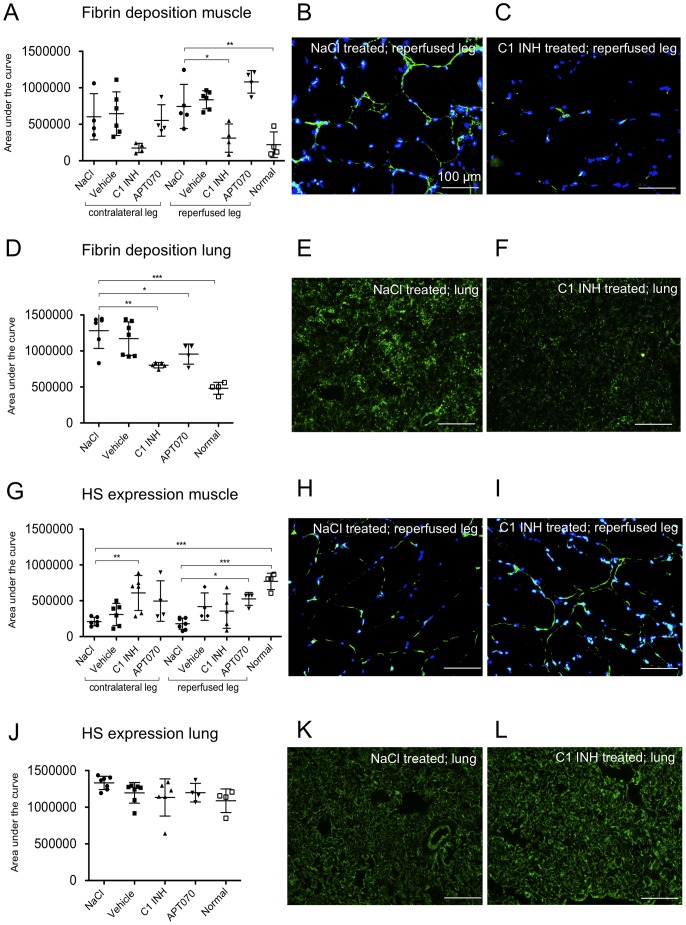
Fibrin deposition as well as heparan sulfate (HS) expression in muscle and lung tissue. (A, D, G and J) Quantification data from immunofluorescence stainings. (B and C) Representative immunofluorescence images for fibrin deposition. Counterstaining with DAPI (blue channel), fibrin visible in the green channel (FITC). E and F, Representative immunofluorescence images for fibrin deposition in lung tissue. (H and **I**; **K** and **L**) Representative immunofluorescence images of HS expression in muscle and lung, respectively. HS visible in the green channel (FITC). One-way ANOVA followed by Dunnett's post hoc test for significance vs. NaCl controls was used. Error bars indicate mean ± SD. *P<0.05; **P<0.01; ***P<0.001.

To analyze the involvement of the coagulation system in peripheral IRI and distant lung damage, muscle as well as lung tissue was stained for fibrin deposition (Figure 5A–F). Fibrin deposits were found in the reperfused muscle in the NaCl, vehicle and APT070 treated groups and were significantly reduced by C1 INH (P<0.05) (Figure 5A–C). C1 INH also reduced fibrin deposition in the lung tissue as compared to NaCl control (P<0.01) (Figure 5D–F). The glycocalyx component heparan sulfate (HS) was detected by immunofluorescence staining (Figure 5G–L). Reduced HS expression was found in the contralateral and reperfused muscle of NaCl controls. C1 INH-treated rats showed significantly preserved expression of HS in tissue of the contralateral muscle as compared to the NaCl control group (P<0.01). However, in the reperfused muscle no differences could be detected between the C1 INH and NaCl groups (Figure 5G–I). APT070 treated rats showed significantly preserved expression of HS in the reperfused muscle in comparison to NaCl treated rats (P<0.05). No inter-group differences were found for HS expression in lung tissue (Figure 5J–L).

### Effect of C1 INH treatment on apoptosis in muscle and lung tissue

**Figure 6:**
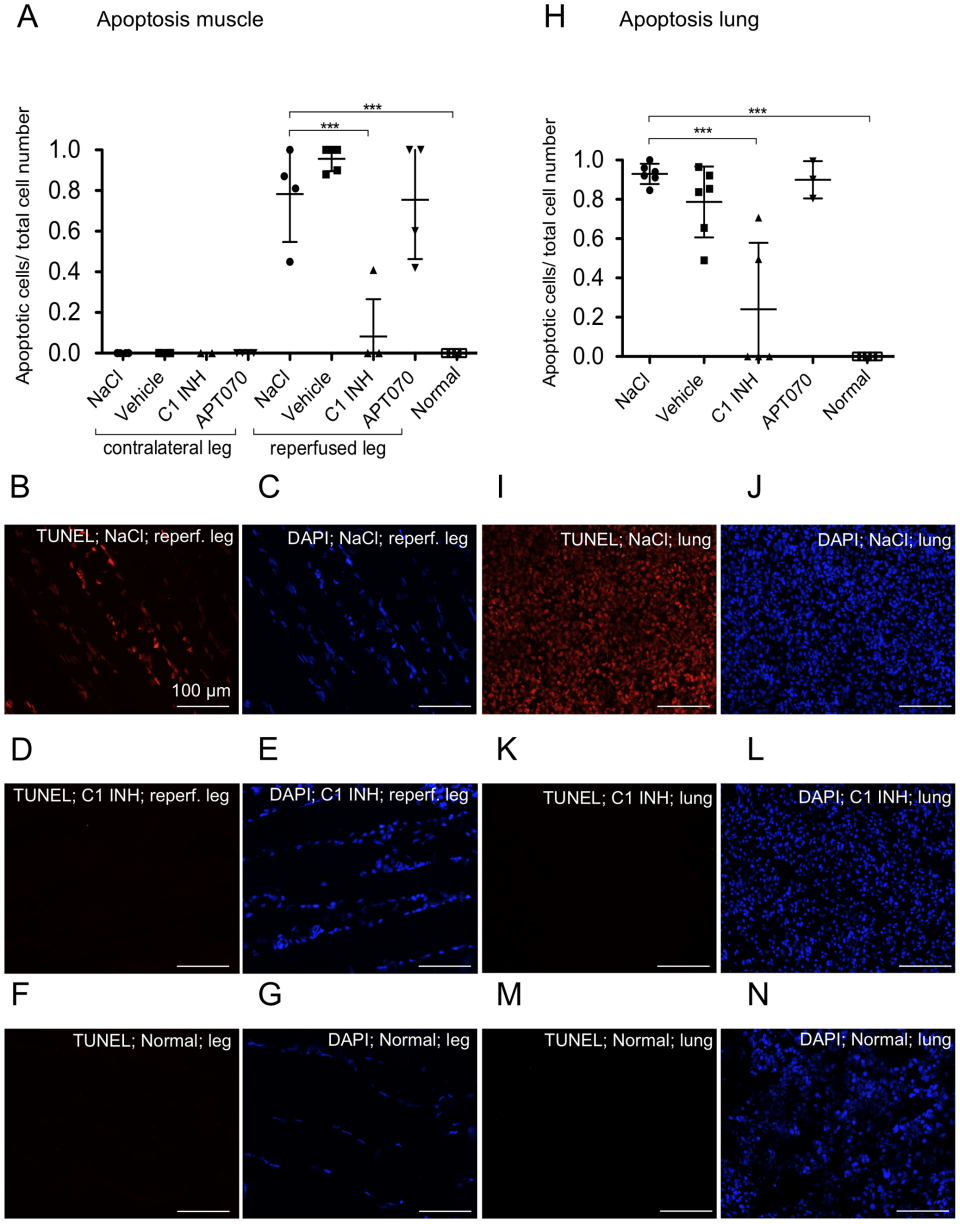
Frequency of apoptotic cells in muscle and lung tissue. (A and H) Quantitative analysis of TUNEL staining in muscle and lung tissue, respectively. (**B–G**) and (**I–N**) Representative immunofluorescence images of TUNEL staining of reperfused muscle and lung, respectively. TUNEL-positive cells are shown in red (**B, D, F, I, K, M**), corresponding DAPI staining of all nuclei in blue (**C, E, G, J, L, N**). One-way ANOVA followed by Dunnett's post hoc test for significance vs. NaCl controls was used. Error bars indicate mean ± SD. *P<0.05; **P<0.01; ***P<0.001.

Apoptosis was measured using the TUNEL assay. The ratio of TUNEL-positive cells to total cell number was calculated. Whereas in the contralateral muscle no apoptotic cells were detected, cells in the reperfused muscle of the NaCl (0.78±0.24), vehicle (0.96±0.06) and APT070 (0.76±0.29) treated groups showed a high degree of apoptosis. C1 INH (0.08±0.18), but not APT070, treatment led to a significant reduction of apoptotic cells (P<0.001) in the reperfused muscle (Figure 6A–G). Similar results were found for lung tissue, where C1 INH treated rats (0.24±0.34) also showed significantly less apoptosis as compared to the NaCl (0.93±0.05), vehicle (0.79±0.18) and APT070 (0.90±0.10) (Figure 6H–N).

### Effect of C1 INH treatment on expression of bradykinin receptor b1 as well as b2 in lung tissue

**Figure 7:**
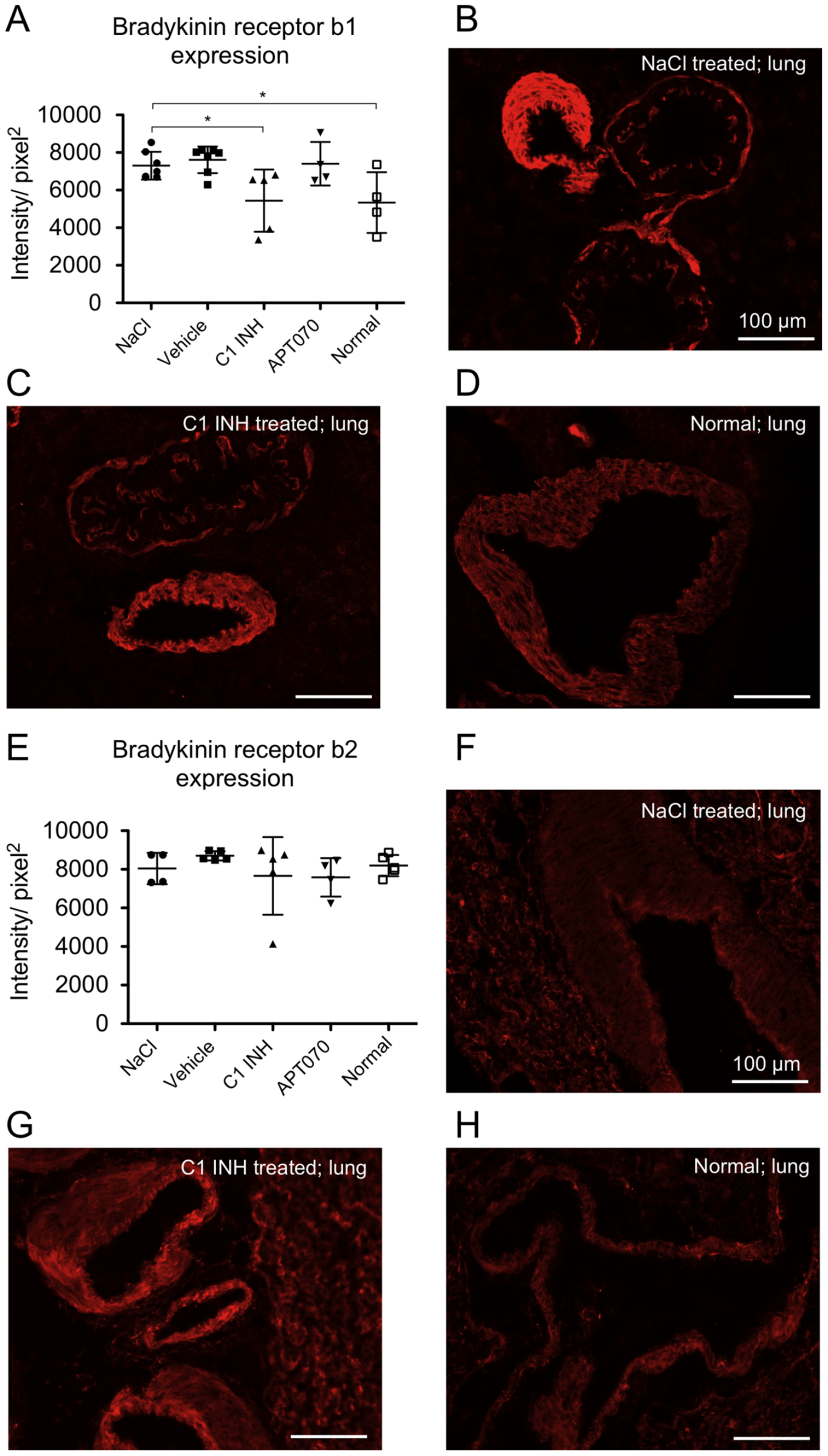
Endothelial expression of bradykinin receptor b1 as well as b2 in lung tissue. (A and E) Quantification data from immunofluorescence stainings. (B–D) Representative immunofluorescence images of bradykinin receptor b1 as well as (**F**–**H**) bradykinin receptor b2 staining in vessels of lung tissue. One-way ANOVA followed by Dunnett's post hoc test for significance vs. NaCl controls was used. Error bars indicate mean ± SD. *P<0.05; **P<0.01; ***P<0.001.

Lung sections were stained for bradykinin receptor b1 (Figure 7 A–D) as well as b2 (Figure 7E–H). Specificity of bradykinin receptor staining was verified by competitive inhibition with the respective b1- or b2-peptides (data not shown). In contrast to APT070, C1 INH inhibited up-regulation of bradykinin receptor b1 in lung tissue as compared to control groups (P<0.05 vs. NaCl). No inter-group differences were found for receptor bradykinin receptor b2 expression.

### Analysis of infiltration of myeloperoxidase positive cells and expression of VE-cadherin in lung tissue

**Figure 8:**
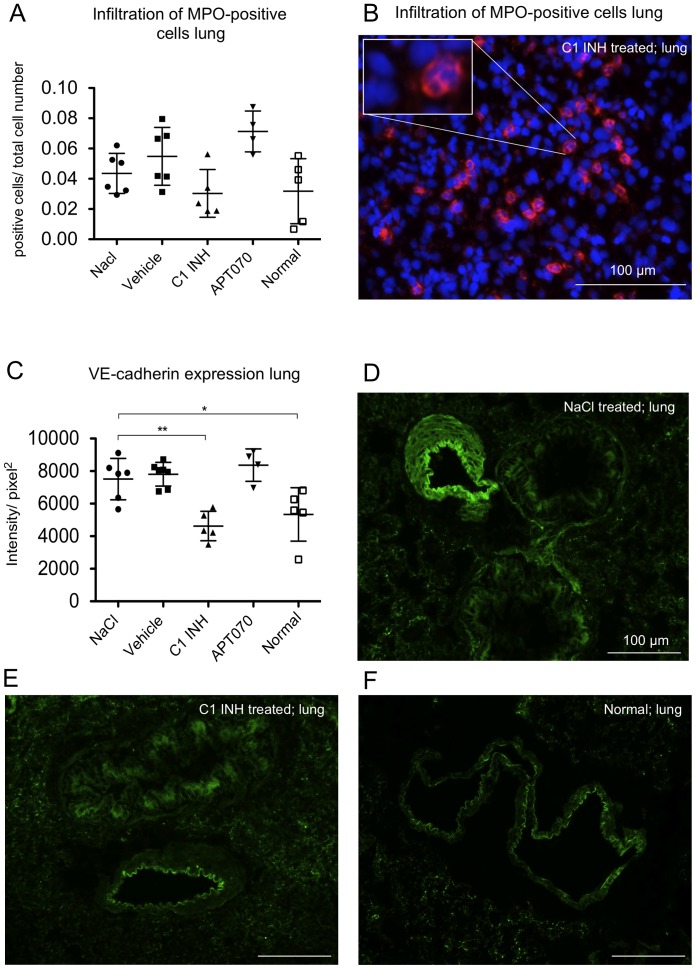
Infiltration of myeloperoxidase positive cells in lung tissue as well as VE-cadherin expression. (A) Quantitative evaluation and (**B**) representative immunofluorescence image of MPO expression in lung tissue. The blue channel shows DAPI staining, the red channel (CY3) shows MPO positive cells. (C) Quantification data from immunofluorescence stainings of VE-cadherin. (D–F) Representative images of VE-cadherin staining. One-way ANOVA followed by Dunnett's post hoc test for significance vs. NaCl controls was used. Error bars indicate mean ± SD. *P<0.05; **P<0.01; ***P<0.001.

Infiltration of pro-inflammatory cells such as neutrophil granulocytes was assessed by immunofluorescence staining for myeloperoxidase. No significant differences between NaCl controls and the other treatment groups could be observed. However, C1 INH treated rats showed a trend for reduction of MPO-positive cells (Figure 8A, B). Analysis of the expression of VE-cadherin, a protein important for the endothelial barrier function, in lung tissue showed an up-regulation in the NaCl control group as compared to normal rats. Up-regulation of VE-cadherin was prevented in C1 INH treated rats (P<0.01) (Figure 8C–F).

### Analysis of plasma levels of cytokines, chemokines and growth factors after 24 h reperfusion

Multiplex suspension array technology was used to quantify levels of different cytokines, chemokines and growth factors in EDTA-plasma taken after 24 h of reperfusion. Analysis revealed that C1 INH treatment significantly reduced levels of Interleukins (IL) IL-1α, IL-7, IL-17 and IL-18 as well as IFN-γ, MIP-1α (macrophage inflammatory protein, CCL3), MIP-3α (CCL20) and TNF-α (P<0.05). EPO (erythropoietin), CXCL1, RANTES (regulated and normal T cell expressed and secreted, CCl5), VEGF (vascular endothelial growth factor), IL-4, IL-5, IL-10, MCP-1 (monocyte chemotactic protein 1) and M-CSF (macrophage colony-stimulating factor) were not affected. Data are expressed as means ± SD. IL-1beta, IL-2, IL-6, IL12p70, IL-13, G-CSF, GM-CSF were below detection level and are not listed (Table 1).

**Table 1:** Plasma levels of cytokines, chemokines and growth factors (in pg/ml) at baseline and after 24 h of reperfusion.

Marker^†^	Baseline	NaCl	C1 INH
EPO	278.9±183.2	729.0±427.7	346.9±353.1
CXCL1	93.0±14.5	127.4±77.0	78.3±68.8
IFN-γ	57.6±34.9	120.3±100.2	19.1±11.4*
IL-1α	9.0±4.9**	83.1±44.9	16.9±10.3*
IL-4	22.6±11.4	57.5±39.3	8.3±4.2
IL-5	96.5±11.2	172.7±73.6	108.3±23.7
IL-7	69.1±22.9**	240.9±98.3	67.2±42.0**
IL-10	306.6±51.3	776.3±508.0	279.1±94.2
IL-17A	7.0±1.5**	27.1±14.0	8.3±3.4**
IL-18	1103.0±720.6**	4155.0±1390.0	1115.0± 80.4**
MCP-1	425.0±58.0*	1693.0±982.4	1905.0±638.3
MIP-1α	1097.0±968.0*	4629.0±3045.0	1203.0±762.0*
MIP-3α	10.0±8.6**	48.0 ± 20.2	12.0±9.5**
RANTES	164.0±89.1	310.2±309.7	389.0±442.7
TNF-α	19.1±6.1	40.4±24.9	9.1±7.5*
VEGF	12.0±4.9	16.0±6.0	11.4±2.0
M-CSF	293.6±50.8	381.2±117.1	459.1±79.2

EPO indicates Erythropoietin; CXCL1, Chemokine (C-X-C motif) Ligand 1; IFN-gamma, Interferon-gamma; IL, Interleukin; MCP-1, Monocyte chemotactic protein-1; MIP, Macrophage inflammatory protein; RANTES, Regulated and normal T cell expressed and secreted; TNF-α, Tumor necrosis factor-α; VEGF, Vascular endothelial growth factor; M-CSF, Macrophage colony-stimulating factor. Values are mean ± SD. P<0.05*; P<0.01** by ANOVA with Dunnett's post test vs. NaCl. Multiplex analysis of the shown markers was performed using a standard rat 24-plex panel from Bio-Rad. ^†^IL-1β, IL-2, IL-6, IL12p70, IL-13, G-CSF, GM-CSF were below detection level and are not listed.

## Discussion

The present study aimed to investigate the effects of C1 INH treatment on peripheral IRI and related remote lung damage. Originally, the application of C1 INH was described in the potentially life-threatening disease hereditary angioedema (HAE) [30]. Lower extremity IRI is associated with edema formation in the affected tissue, which is multifactorial and results amongst others from increased vascular permeability [31]. It is also known that limb ischemia may cause distant lung damage, including pulmonary pathology with fibrin-rich microthrombus formation, vascular congestion and pulmonary edema [32]. In the present study we show that C1 INH protected from peripheral IRI by reduction of skeletal muscle edema and maintenance of muscle cell viability. In addition, lung edema formation was prevented by C1 INH treatment. Edema formation in muscle as well as lung tissue required reperfusion. Rats subjected to ischemia only did not show gastrocnemic muscle or lung edema (histologically assessed, data not shown), suggesting that local, humoral or cellular components within the reperfused limb were responsible for mediating distant lung damage [15].

In order to investigate the mechanisms of edema reduction and improvement of muscle viability, we analyzed the involvement of the complement, coagulation and kinin systems since all three systems play important roles in IRI pathophysiology [9], [20], [33]. First, the effect of C1 INH on binding of natural IgG as well as IgM antibodies was determined by immunofluorescence. Indeed, as compared to normal control rats, no significant increase of antibody deposition was found in contralateral muscle tissue as well as in lung. In reperfused muscle we detected high deposition of IgG as well as IgM in NaCl and vehicle treated groups, which was significantly reduced by treatment with C1 INH and APT070. Both are inhibitors of the complement system, but their evident direct effect on natural antibody binding has not been described so far.

In order to assess which complement pathways were mainly affected by C1 INH treatment, deposition of C3b/c (all pathways), factor B (alternative pathway), MBL (lectin pathway), C1q (classical pathway) as well as C4b/c (classical and lectin pathway) were investigated. Previous studies which analyzed the effect of C1 INH treatment on peripheral IRI did not investigate deposition of complement components at all or only as hemolytic C3 and C4 titers [34]. In our study, we showed that deposition of complement components C4b/c and MBL was not increased by peripheral IRI. However, an increased binding of C1q, C3b/c and factor B was found in the contralateral as well as in the reperfused leg, but was not significantly reduced by C1 INH treatment in the reperfused leg. Also in lung tissue C1 INH showed no significant effects on C3b/c and factor B deposition. In contrast, the specific complement inhibitor APT070 significantly reduced deposition of C1q and C3b/c in the reperfused and contralateral legs as well as C3b/c in the lung, while not preventing edema formation or increasing tissue viability. This finding was unexpected as beneficial effects of APT070 were shown earlier for remote and systemic injury following intestinal ischemia and reperfusion in rats and myocardial reperfusion injury in pigs [35],[20]. That APT070 indeed prevented complement activation was also confirmed in vitro by CH50 test as well as cell ELISA and cytotoxicity assay with porcine cells and human serum (data not shown). Based on the above mentioned data we conclude that the beneficial effects of C1 INH treatment were not primarily due to inhibition of the complement system.

Systemic circulation of activated complement components has been shown in models of IRI and deposition of such components on the endothelium of distant organs and tissues may therefore play a role [36]. Another possibility would be that locally produced bradykinin may lead to distant edema formation in the lung once reperfusion starts. In our study, C1 INH treatment led to reduced fibrin deposition in muscle as well as lung tissue. This finding is in line with C1 INH being the main inhibitor of coagulation factors XIa as well as XIIa [21]. Also in a mouse model of stroke it was recently demonstrated that C1 INH treatment reduced intracerebral fibrin formation [37]. An important mechanism for degradation of fibrin into soluble fibrin degradation products is the fibrinolytic system. It was reported that the fibrinolytic pathway can be initiated via direct plasminogen activation through tissue plasminogen activator (tPA), kallikrein (KK) or factor XII, which results in the generation of plasmin [38], [39]. However, as C1 INH inhibits plasminogen activators like FXII, KK as well as to a lesser extent tPA and plasmin itself, increased fibrinolysis will probably not be the main reason for reduced fibrin deposition [40], [41]. Rather, C1 INH dependent inhibition of the activation of the coagulation system may be responsible for the observed significant reduction of fibrin deposits.

Furthermore, as a marker of the integrity of the glycocalyx, we analyzed expression of HS in muscle as well as lung tissue. In muscle, shedding of HS was detected as decreased expression in the NaCl and the vehicle group in the reperfused as well as the contralateral leg, but we did not detect any effect of peripheral IRI on HS expression in the lung. All treatment groups, including vehicle, showed intermediate HS expression patterns between NaCl controls (low) and normal rat tissue [42]. Among these, statistical significance for preservation of HS expression was reached for C1 INH in the contralateral and for APT070 in the reperfused leg.

Next to activation of the fibrinolytic system, factor XIIa initiates the intrinsic pathway of coagulation via FXI activation and also the kinin cascade by cleaving plasma prekallikrein, leading to the formation of bradykinin. Active bradykinin binds to its b2 receptor on the surface of endothelial cells, whereas des-arg-9-bradykinin acts on b1 receptors, both causing vasodilation and increased vascular permeability [43]. In contrast to b2 receptors, b1 receptors are not constitutively expressed but are induced by pro-inflammatory cytokines. We found normal levels of the constitutively expressed receptor b2 in all groups. However, compared with normal rats, bradykinin b1 receptor expression was increased in NaCl controls and this up-regulation was prevented by treatment with C1 INH but not by APT070. This finding corresponds with the reduced edema formation in lung tissue found in C1 INH but not in APT070 treated or control rats and with an earlier report showing that blocking of the b1 receptor, but not b2 receptor, diminished brain edema formation in mice [33]. Similar results were also shown for lung as well as intestinal IRI by using bradykinin receptor antagonists to prevent or attenuate IRI [44], [45]. However, reduced expression of b1 receptors could also be attributed to a reduction of pro-inflammatory cytokine levels via C1 INH, as bradykinin receptor expression can be induced through pro-inflammatory cytokine release.

Pulmonary damage secondary to local IRI can result from embolism but also from circulatory distribution of inflammatory mediators locally produced in the affected tissue [46]. There is no evidence, that exclusively bradykinin is responsible for edema formation in lung in the present model. It was reported that locally produced humoral mediators can cause leukocyte accumulation in lung tissue, which results in pulmonary damage by clogging of the capillaries and release of lysosomal enzymes by leukocytes [15], [47]. Furthermore, an important role in edema formation is attributed to leukotriene B_4_ and other inflammatory mediators, like serotonin or histamine [48], [49].

Two studies reported that C1 INH, via expression of sialyl Lewis^x^ tetrasaccharides and binding to E- and P-selectins, prevents adhesion and migration of leukocytes to the endothelium in vitro as well as in vivo [50], [51]. We analyzed infiltration of MPO-positive cells in lung tissue and indeed a trend, albeit not significant, was found for a reduction of MPO positive cells by C1 INH.

We also analyzed the expression of VE-cadherin, which is a component of adherens junctions of endothelial cells and contributes to their barrier function [52]. In vessels of lung tissue we found elevated levels of VE-cadherin in NaCl, vehicle as well as APT070 treated rats, whereas C1 INH treated rats showed VE-cadherin expression levels similar to normal rats. Currently, not much is known about the mechanisms by which VE-cadherin-mediated cell-cell junctions are regulated. It could be speculated that in the present study the increase of VE-cadherin expression could be due to repair mechanisms, whereas C1 INH maintains endothelial cell integrity and avoids activation of these mechanisms [53]. However, a more detailed analysis of the mechanism of VE-cadherin regulation in IRI would be necessary to support this hypothesis, which is beyond the scope of the present study.

In IRI apoptosis plays an important pathophysiological role and is an event of reperfusion, as it requires energy and is associated with cell shrinkage and phagocytosis without loss of membrane integrity [54]. In our study, C1 INH treated rats showed significantly less apoptosis as compared to the NaCl control group. These data confirm earlier reports describing that C1 INH improves the outcome of myocardial IRI via anti-apoptotic activity independent of its serine protease inhibitory activity by normalization of ratio of the Bcl-2/Bax expression [55]. Furthermore, it was shown that C1 INH reduced infarction size in a mouse model of myocardial infarction via inhibition of leukocyte transmigration into the ischemic tissue, which is also not mediated through its protease activity [56].

The systemic inflammatory response, which is initiated in IRI is characterized by the release of pro-inflammatory cytokines, like TNF-α [57]. Our results demonstrated that C1 INH treatment led to significantly reduced levels of several pro-inflammatory cytokines. In a model of myocardial IRI it was shown that IL-17A plays a pathogenic role by inducing cardiomyocyte apoptosis and neutrophil infiltration [58]. We found reduced plasma levels of IL-17A in C1 INH treated rats, which fits with the observed reduction of apoptosis in muscle and lung tissue by C1 INH treatment. Also MIP-1α plays an important role in mediating an acute inflammatory response – another chemokine that was significantly reduced in C1 INH treated rats in our study [59].

In 2004, Inderbitzin and colleagues presented a study of transgenic mice overexpressing human C1 INH (plasma levels of 1–2 mg/ml), which were used for a lower torso IRI model. They found that muscle as well as lung tissue was protected from endothelial cell damage by measuring the amount of extravasation of ^125^I-labelled albumin, reflecting a direct functional measurement of endothelial integrity [53]. We showed here for the first time in non-transgenic animals that C1 INH at a low, clinically applicable dose of 50 IU/kg significantly reduced peripheral IRI in muscle and, in particular, that also lung injury was significantly reduced.

In conclusion, C1 INH is a multifaceted protein, which acts on multiple inflammatory cascades relevant in IRI pathology. Via inhibition of kallikrein, FXIa, FXIIa as well as the complement system, it regulates IRI associated inflammatory and thrombotic processes. Our data support the regulatory effect of C1 INH on the coagulation- and the kinin system in IRI. A very potent inhibitory effect of human C1 INH on edema formation and apoptosis in skeletal muscle as well as in lung was observed. In addition, the up-regulation of bradykinin receptor b1 was prevented by C1 INH. These results may be a hint that C1 INH plays an important role in inhibition of the kinin system in this animal model of hind limb IRI. Furthermore, C1 INH also prevented fibrin deposition. Analysis of the effect of C1 INH on the complement cascades revealed that C1 INH reduced peripheral IRI not primarily by inhibition of the complement system. This conclusion is supported by APT070 data, which showed a significant reduction of C1q and C3b/c in the reperfused leg, but did not reduce edema formation in muscle and lung tissue. Furthermore, C1 INH reduced plasma levels of IFN-γ, IL-1α, IL-7, IL-17A, IL-18, MIP-1α, MIP-3α and TNF-α. All in all, C1 INH may provide a promising therapy to reduce peripheral IRI as well as distant lung injury in complicated and prolonged surgical interventions requiring tourniquet application.
